# Feasibility of ^129^I groundwater dating calibrated by both ^81^Kr and ^4^He for the assessment of deep geological repositories in Japan

**DOI:** 10.1038/s41598-024-66250-3

**Published:** 2024-07-08

**Authors:** Tomoko Ohta, Takuma Hasegawa, Wei Jiang, Guo Min Yang, Zheng-Tian Lu, Yasunori Mahara

**Affiliations:** 1 Civil Engineering Research Laboratory (Sustainable System Research Laboratory), Central Research Institute of Electric Power Industry, Abiko, Chiba 270-1176 Japan; 2 Department of Nuclear Technology, Nagaoka University of Technology, Kamitomioka, Nagaoka, Niigata, 940-2188 Japan; 3 CAS Center for Excellence in Quantum Information and Quantum Physics, School of Physical Sciences, University of Science and Technology of China, Hefei, 230026 China; 4 Hefei National Laboratory, University of Science and Technology of China, Hefei, 230088 China; 5 Kyoto University, Sakyo-Ku, Kyoto, 606-8501 Japan

**Keywords:** Environmental sciences, Hydrology

## Abstract

Iodine-129, which is a promising tracer for dating old groundwater, has been used as a tracer for deep upwelling groundwater. The nuclide is expected to be one of the key factors for site selection for high-level radioactive waste disposal, which is a global societal issue. The pre-anthropogenic ^129^I/^127^I ratio for marine iodine is (1.50 ± 0.15) × 10^−12^, which could be considered the initial value for ^129^I dating. This study identifies the challenges in groundwater age dating using ^129^I/^127^I. We measured the ratios of ^129^I/^127^I and ^81^Kr/Kr and concentration of ^4^He in groundwater from boreholes on the northern coast of Japan. The ^129^I dating results were not coincident with the other groundwater dating results. The iodine in the groundwater was inferred to be released in situ from marine organisms in sediments of various ages. We estimated that the primordial iodine ratio originating from seawater was ~ 1 × 10^–13^ (8 × 10^–14^ ~ 2 × 10^–13^). The groundwater age deduced from the ^129^I/^127^I ratio using this value agrees with other groundwater dating results.

## Introduction

Limited groundwater mobility is one of the most important criteria for the safe and long-term disposal of high-level radioactive wastes in deep geological formations. Groundwater flow is the most critical factor in this assessment. Such assessments are important in Japan, where deep underground environments are rich in groundwater. An excellent way to determine groundwater mobility is to compare the residence time of the groundwater with the strata depositional age of the basement rocks that formed in the basin^1–3^. Most of the traditional methods for dating old groundwater involve ^36^Cl and ^4^He^4–6^. More recently, the cosmogenic noble gas isotope ^81^Kr (T_1/2_ = 229 ka) has been used to determine the age of groundwater up to 1.3 Ma^4,7–11^.

In addition, the presence of groundwater rising to the surface from deep underground indicates that a particular location is not appropriate for a repository site. This groundwater upwelling provides a pathway for radionuclides buried deep underground to move to the surface, where the nuclides could then contaminate human residential areas. This upwelling phenomenon is easily understood in cases where groundwater rises to the surface from deep underground and is visible at the surface. However, it is not clear whether groundwater in the deeper subsurface has risen from even deeper underground.

Naturally occurring radionuclides in groundwater (^4^He, ^81^Kr, ^36^Cl) may be able to show whether the groundwater is older than the sedimentary strata deposited in a basin; therefore, these radionuclides can be used to determine whether the water that wells up toward the ground surface at a potential disposal site is groundwater with a long residence time. When ^81^Kr is not detected and ^4^He accumulation is extremely high in groundwater (corresponding to a groundwater age longer than a strata depositional age of 1 Ma), the groundwater may flow from great depth. Dissolved ^129^I in groundwater is a long-lived cosmogenic radionuclide (T_1/2_ = 15.7 Ma), and the ^129^I/^127^I ratio in groundwater has been used to estimate the residence time of groundwater with ages of tens of Ma^12–14^. Due to its long half-life, ^129^I cannot be used as a tracer for groundwater with ages less than 5 Ma, while it might be a potential age tracer for groundwater with a circulation history longer than 10 Ma. By combining ^81^Kr, ^4^He and ^129^I, it might be possible to verify the presence of groundwater with ages of tens of ka to 100 Ma.

Several studies have estimated groundwater ages of several tens of Ma by ^129^I groundwater dating at the Kanto gas field site^12^, the Horonobe site^13^, and the Kyushu site^14^ in Japan. In another study in Japan, the residence time of groundwater in the Kanto gas field estimated by the ^36^Cl and ^4^He dating methods coincided with the strata depositional age of the sedimentary rock where groundwater was stored^2^. On the other hand, the residence time estimated by the ^129^I dating method significantly differed from the dating results^2^ based on the ^36^Cl and ^4^He concentrations. The pre-anthropogenic ^129^I/^127^I ratio in marine iodine has typically been reported to be (1.50 ± 0.15) × 10^−12^, which could be considered the initial input value for ^129^I groundwater dating^15,16^. Information on past variations in isotopic ratios in seawater is stored in coral^17,18^ and algae samples^15,19,20^. Moran et al.^16^ reported the iodine isotopic ratio of old seawater from deep marine sediments. Furthermore, the ^129^I/^127^I isotopic ratio in seawater estimated from pre-World War II algae was 1.5 × 10^–12^ based on a mean of multiple algae samples collected from the pre-thermonuclear test era^15^. The ^129^I/^127^I isotopic ratio in coral was observed to be on the order of 10^–12^ before 1945^21^. On the other hand, the ^129^I/^127^I isotopic ratios of algae samples collected before World War II and stored in museums have been observed to be on the order of 10^–13^ or more^15,19^. The observed ^129^I/^127^I isotopic ratios in samples are widely divergent. The typical ^129^I/^127^I isotopic ratios of natural sources are between 10^–12^ (Kilius et al.^22^) and 6 × 10^–13^ (Fabryka-Martin et al.^23^). Previous studies based on ^36^Cl (Mahara et al.^1^) and ^4^He (Mahara et al.^2^) in groundwater^1^ predicted that the primordial ^129^I/^127^I ratio in groundwater ranges from 1.4 × 10^–13^ to 6 × 10^–13^ based on fossil seawater collected around the Kanto gas field in Japan.

**Figure 1:**
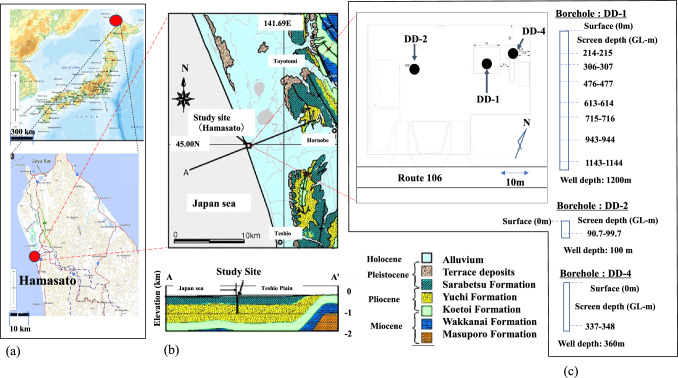
Sampling site and geological map. (**a**) Geospatial Information Authority of Japan (GIS), (**b**) geological map and geological cross section of the site from Ikawa et al. ^39^, (**c**) location of three boreholes (DD-1, DD-2, DD-4) and screen depth of the boreholes. The edited map was obtained from the original map (https://www.gsi.go.jp/tizu-kutyu.html). The copyright holder of the map is the Geospatial Information Authority of Japan.

In this paper, we determine the primordial value of ^129^I/^127^I in groundwater originating from fossil seawater and meteoric water in coastal sedimentary areas to address the societal challenge of site selection for geological disposal. We proposed the primordial ^129^I/^127^I ratio using the following methods: (1) an absolute groundwater dating method using dissolved ^81^Kr concentrations compiled from other dating methods based on ^3^H and ^14^C radioactivity and the dissolved noble gas concentration in deep groundwater collected from boreholes drilled in northern coastal Japan and (2) a new method focusing on biogenic elements (B, Br and C (TOC)) in seawater. The study site is defined as a sedimentary coastal area that sank into the deep sea in the pre-early Pleistocene (Fig. 1 and Fig. S1).

## Results

### Groundwater recharge age estimated from the radioactivity of ^81^Kr and ^14^C and the dissolved ^4^He concentration

**Figure 2:**
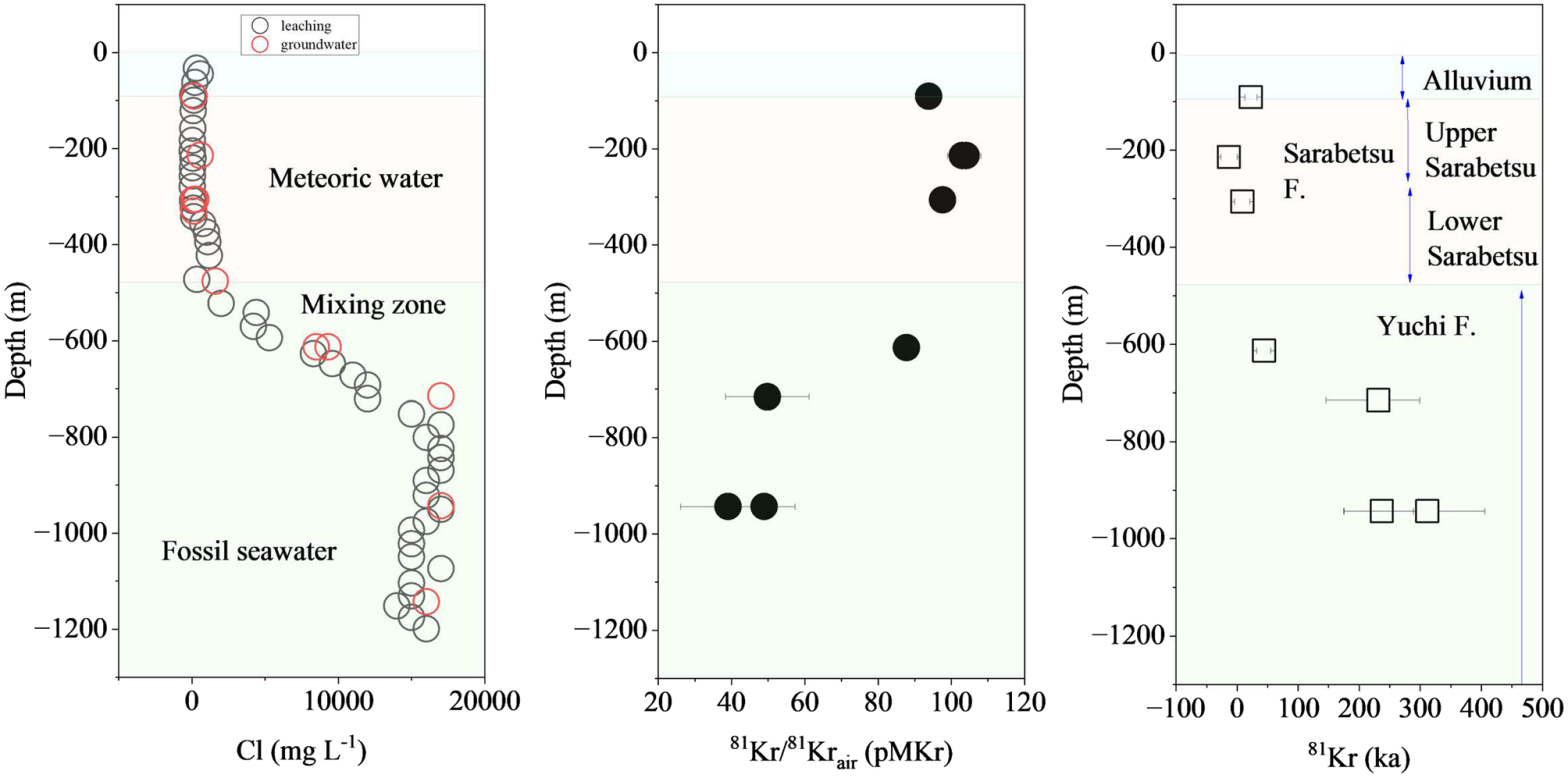
Concentration depth profiles of Cl, ^81^Kr, and estimated recharge age of groundwater. 〇: concentration of Cl (red circle: groundwater, black circle: pore water), ●: concentration of ^81^Kr, □: recharge age of groundwater estimated from ^81^Kr

The study site was estimated to be subseafloor in the past, and coastal sediments continued to be deposited on the sea floor after the deposited sediments finally emerged from the sea surface (see Methods and Fig. S1). Supplementary Fig. S2 shows the major ions in the groundwater, which indicate that freshwater was distributed between the alluvium and Sarabetsu Formations and that brine included the freshwater distributed beneath the Yuchi Formation in the mixing zone. Based on the vertical distribution of the Cl concentration in the groundwater, meteoric water infiltrated the Upper Yuchi Formation, and the original seawater was replaced with recharged meteoric water (Fig. S2). The tritium concentration in the groundwater samples was below the detection limit at depths ranging from 90 to 1143 m, indicating that the recharge age of the groundwater in each formation was greater than 60 a (Table S1). Table 1 lists the concentrations of ^81^Kr and ^85^Kr in the groundwater and the corrected ^81^Kr concentration (pMKr, percent modern Kr). The corrected concentration of ^81^Kr in the groundwater ranged from 39 to 97.6 pMKr at depths ranging from 90.7 m to 99.7 m in the Upper Sarabetsu Formation and below 306 m between the Lower Sarabetsu and Lower Yuchi Formations. The ^81^Kr in the groundwater at depths from 214 to 215 m in the Upper Sarabetsu Formation was 103–104 pMKr. Figure 2 shows the vertical profile of the concentrations of Cl and ^81^Kr dissolved in the groundwater and the exchange period of seawater with meteoric water deduced from the ^81^Kr dating results. The concentration of ^81^Kr in the groundwater in the Sarabetsu Formation was 94–98 pMKr from a depth of 90 m to 307 m, corresponding to a residence time of 9–22 ka. The presence of meteoric water has already been validated in the formation, considering that the recharge age of meteoric water is less than the estimated 22 ka based on ^81^Kr dating of the groundwater. The concentrations of ^14^C and ^4^He in the groundwater from a depth of 90–307 m in the borehole ranged from 15 to 22 pMC and from 6 × 10^–8^ to 10 × 10^–8^ cc STP g^-1^ (STP, standard temperature pressure), respectively (Tables 1 and S1). The estimated groundwater ages at the same depth ranged from 8 to 13 ka based on ^14^C and from 18 to 34 ka based on ^4^He^24^. The recharge era of these meteoric waters estimated by the ^81^Kr dating results was roughly in line with the ages estimated from both the dissolved concentrations of ^14^C and ^4^He in the Sarabetsu Formation. The results estimated from the ^81^Kr, ^14^C and dissolved ^4^He concentration dating methods clearly showed that the groundwater found in the Sarabetsu Formation originated from meteoric water discharged approximately 22 ± 10 ka.

**Table 1:** Concentrations of ^81^Kr, ^85^Kr, and ^4^He in the groundwater and estimated recharge age of the groundwater.

Geology							Measurement value								Age upper limit (90% confidence level) ka	^4^He** cc STP g^-1^ (recharge age)
	Sedimentary age Ma				Borehole								^81^Kr age recharge age ka			
		Depth m				Sampling date	^85^Kr dpm ccKr^-1^	^81^Kr pMKr	^81^Kr/^81^Kr_air (pMKr)				err +	err−		
Alluvium	~ 0.02	0	–	90		–	–	–		–		–				–
Upper Sarabetsu F	1.3*	90.7	–	99.7	DD-1	Oct., 3, 2017	5.94 ± 0.41	94.3 ± 2.5	93.81	±	2.72	22.3	9.8	− 9.9	< 10.8	6–10 × 10^–8^ (0.018–0.034 Ma)
		214	–	215	DD-2	Oct., 23, 2017	0.9 ± 0.3	104 ± 4	104	±	4.05	− 13.5	13.5	− 13.5	< 13.1	
		214	–	215	DD-2	Oct., 23, 2017	1.0 ± 0.2	103 ± 4	103	±	4.05	− 10.1	13.5	− 13.4	< 28.4	
Lower Sarabetsu		306	–	307	DD-1	Jun. 7, 2017	3.19 ± 0.41	97.7 ± 3.4	97.6	±	3.55	8.5	13	− 12.3		
Upper Yuchi F	2*	613	–	614	DD-1	Sep., 13, 2017	1.7 ± 0.3	88 ± 3	87.7	±	3.1	44.1	12.0	− 11.0		2–9 × 10^–6^ (1-2 Ma)
		715	–	716	DD-1	May 21, 2017	33.2 ± 3.9	72.0 ± 5.8	49.8	±	11.4	231	86	− 68		
Lower Yuchi F		943	–	944	DD-1	Nov., 16, 2017	38.3 ± 1.3	75 ± 4	48.9	±	8.4	237	62	− 52		
		943	–	944	DD-1	Nov., 16, 2017	38.1 ± 2.5	70 ± 6	39.0	±	12.9	312	137	− 94		
		943	–	944	DD-1	Nov., 17, 2017	NA	NA								

*Ikawa et al.^39^

**in-situ sampling from 214 to 944m, GL sampling above 200m (Hasegawa et al.^24^)

The concentration of ^81^Kr dissolved in the groundwater in the Upper Yuchi Formation, corresponding to the mixing zone between freshwater and brine (connected with seawater), was lower than that in the Lower Sarabetsu Formation. The ^81^Kr concentration in the Upper Yuichi Formation decreased with increasing concentrations of Cl and Br, and the δD value also increased with increasing δ^18^O value. Finally, the estimated ^81^Kr age increased with increasing groundwater sampling depth in the formation. Although the Cl concentrations in the groundwater and the extracted porewater were constant at 17,000 mg L^-1^ below 715 m depth, the ^81^Kr dating ages were also almost constant at 0.16–0.45 Ma. Assuming that seawater was trapped as pore water before 2 Ma when ancient seawater permeated the Yuchi Formation (Fig. S1), the detection of ^81^Kr dissolved in the seawater that intruded into rock pores can be difficult as radioactive decay proceeded for 2 Ma. The presence of groundwater with a Cl concentration (17,000 mg L^-1^) lower than that of seawater could be due to groundwater dilution by water released from the dehydration of organic matter and minerals^25^. Alternatively, if brackish water from lagoons and/or rivers permeated into the Yuchi Formation approximately 0.15–1 Ma, this permeation might explain the lower Cl concentration relative to that of modern seawater (e.g., the salinity of Tokyo Bay is 22–34‰ with an average of approximately 32‰)^26,27^. The ^81^Kr concentration in the groundwater in both the Upper and Lower Yuchi Formations suggested that the age of the seawater and/or brackish water from the shallow bay and lagoon that intruded into the Yuchi Formations was at least 0.31 Ma (at most 0.45 Ma). Dissolved ^4^He in the groundwater was collected by in situ sampling (Fig. S3b,c). Unlike typical sampling methods based on groundwater pumping, this method prevents the sampled groundwater from degassing and protects it from contamination by the modern atmosphere. The concentration of ^4^He dissolved in the groundwater collected in situ from the Yuchi Formation was on the order of 10^–6^ cc STP g^-1^ (2–9 × 10^–6^ cc STP g^-1^), which corresponded to mud pores where ^4^He was produced in situ and accumulated in the host sedimentary rock. The accumulated amount of ^4^He in the groundwater indicates an accumulation time of 1–2 Ma, which is the estimated residence time of the groundwater^24^, and the age estimated from this ^4^He accumulation rate roughly corresponds to the geological sedimentary age of the transformation from a deep bay environment to a shallow lagoon environment. The groundwater in the Yuchi Formation originated from fossil seawater, the brackish water environment changed from a deep bay environment to a shallow lagoon environment in less than 2 Ma, and meteoric water intruded before 0.04 Ma and was found at 614 m in the formation.

### Groundwater recharge age estimated from the ^129^I/^127^I ratio in groundwater

**Figure 3:**
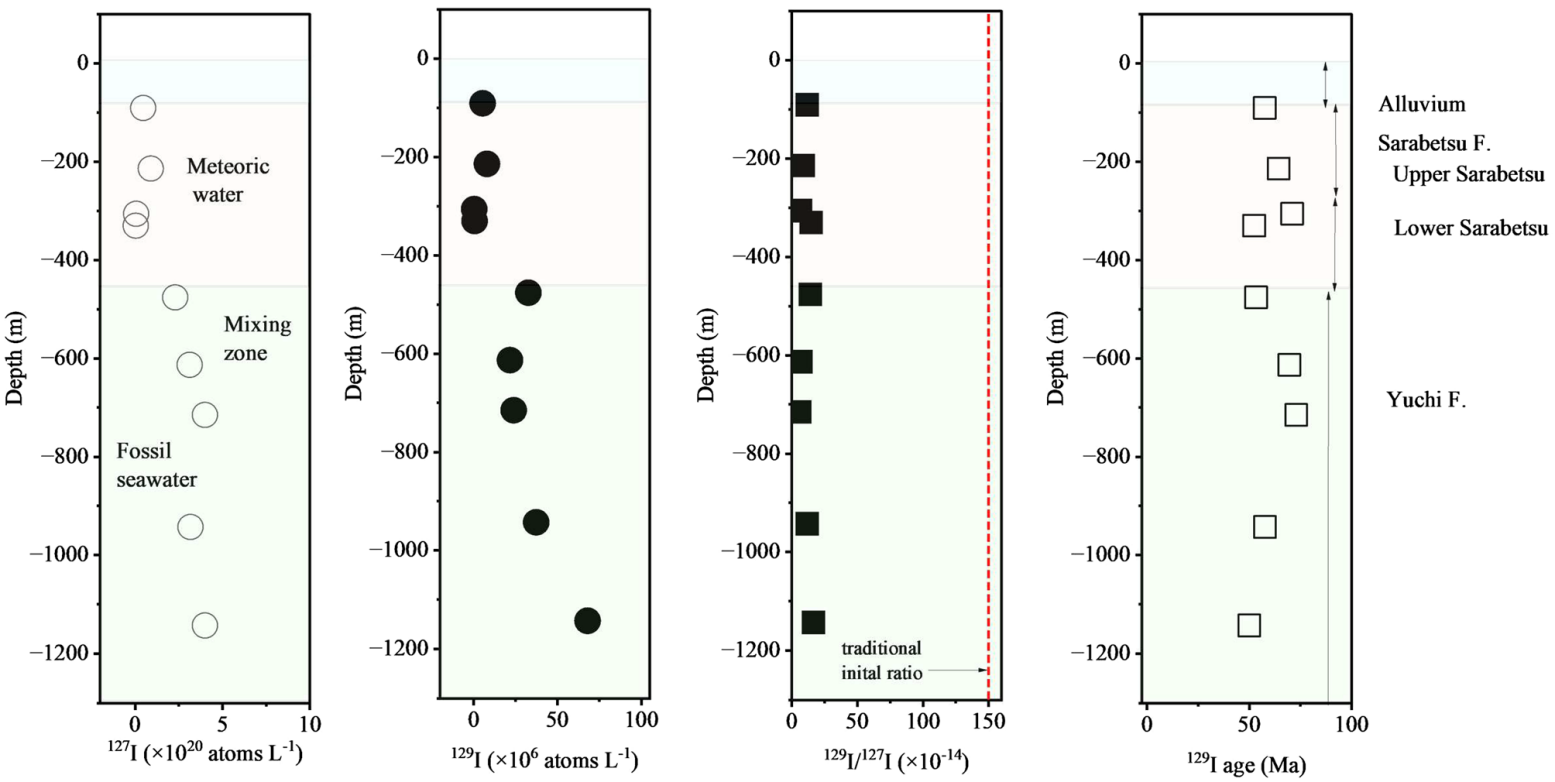
Concentration depth profiles of ^127^I, ^129^I, and ^129^I/^127^I ratio in groundwater and the residence time of groundwater based on the traditional ^129^I groundwater dating method. 〇: concentration of ^127^I in groundwater, ●: concentration of ^129^I in groundwater, ■: ^129^I/^127^I ratio in groundwater, □: recharge age of groundwater estimated from traditional ^129^I dating, ----: traditional initial ratio (1.5 × 10^–12^ by Fehn et al. ^15^).

The concentrations of ^127^I and ^129^I and the isotopic ratios of ^129^I/^127^I ranged from 0.9 to 84 mg L^-1^, from 4 × 10^5^ to 7 × 10^7^ atoms L^-1^, and from 6 × 10^–14^ to 17 × 10^–14^, respectively (Table 2). Figure 3 shows the concentration versus depth profiles of ^127^I (atoms L^-1^), ^129^I (atoms L^-1^), and ^129^I/^127^I (atomic ratio) and the deduced ^129^I age of the groundwater estimated from the traditionally accepted initial ratio of ^129^I/^127^I (1.5 × 10^–12^). The ^129^I/^127^I ratios in the groundwater were generally on the order of 10^–13^. The estimated ^129^I age based on the initial ratio of 1.5 × 10^–12^ ranged from 50 to 71 Ma. Table 3 shows a comparison of the recharge ages of groundwater samples from 3 tracer methods: the ^81^Kr, ^4^He and traditional ^129^I groundwater dating methods. The estimated recharge age of the groundwater deduced via the ^129^I dating method showed large discrepancies of tens of Ma with the strata depositional age at the observation site. We calculated the recharge age of groundwater for a given initial value of ^129^I/^127^I (1.5 × 10^–12^~8.80 × 10^–14^). These groundwater ages were 0~23 Ma for 1.65 × 10^–13^, − 8~15 Ma for 1.18 × 10^–13^ and − 14~9 Ma for 8.80 × 10^–14^. These age also did not agree with groundwater age estimated from other groundwater ages.

**Table 2:** Concentrations of ^127^I and ^129^I and the ^129^I/^127^I ratio in the groundwater.

Geology	Depth m			Borehole	^127^I	^127^I	^129^I		^129^I/127_I_	
						× 10^20^	× 10^6^		× 10^–14^	
					mg L^-1^	atoms L^-1^	atoms L^-1^	SD	–	SD
Upper Sarabetsu F	90.7	–	99.7	DD-2	9.6	0.5	5.38	0.86	11.8	1.9
	214	–	215	DD-1	19.0	0.9	7.91	1.23	8.8	1.4
Lower Sarabetsu F	306	–	307	DD-1	1.2	0.1	0.388	0.118	6.55	1.99
	337	–	348	DD-4	0.9	0.0	0.600	0.100	14.8	3.0
Upper Yuchi F	476	–	477	DD-1	48.3	2.3	32.6	1.39	14.2	0.6
	613	–	614	DD-1	66.0	3.1	21.7	4.00	6.9	1.3
	715	–	716	DD-1	84.3	4.0	23.9	3.40	6.0	0.9
Lower Yuchi F	943	–	944	DD-1	66.9	3.2	37.32	2.87	11.8	0.9
	1143	–	1144	DD-1	84.3	4.0	68.0	4.0	16.5	0.5

**Table 3:** Comparison of the residence times of groundwater samples determined from 3 tracer methods: the ^81^Kr, ^4^He and traditional ^129^I groundwater dating methods.

Geology	Sedimentary age Ma	Depth m			^81^Kr age			^4^He age	^129^I age by various initial ratio				
					Ma	err +	err-	Ma	initial ratio	1.5 × 10^–12^ Ma	1.65 × 10^–13^ Ma	1.18 × 10^–13^ Ma	8.80 × 10^–14^ Ma
Alluvium	~ 0.02	0	–	90	–	–		–	–	–	–		
Upper Sarabetsu F		90.7	–	99.7	0.022	0.010	− 0.010	0.018–0.034		58	8	0	− 7*
		214	–	215						64	14	7	0
		214	–	215									
Lower Sarabetsu		306	–	307						71	21	13	7
	1.3	337	–	348						52	2	− 5*	− 12*
Upper Yuchi F	2	476	–	477				1–2		53	3	− 4*	− 11*
		613	–	614	0.04	0.012	− 0.01			70	20	12	5
		715	–	716	0.23	0.086	− 0.07			73	23	15	9
Lower Yuchi F		943	–	944	0.24	0.062	− 0.05			58	8	0	-7*
		943	–	944	0.31	0.137	− 0.09						
		943	–	944	–								
		1143	–	1144	–					50	0	− 8*	− 14*

*Negative values indicate that the initial value is smaller than the isotopic ratio in groundwater.

**The ^129^I/^127^I ratio in groundwater with recharge ages up to 5 Ma is within the error range of the initial values.

### Iodine and biogenic elements

Figure S4 shows the concentrations of ^127^I and ^129^I in the groundwater with respect to TOC content. The ^127^I concentration was correlated with TOC (correlation coefficient (R^2^) = 0.819, *p*-value (*P*) = 0.000797 < 0.05). However, the ^129^I concentration was not correlated with the TOC content (R^2^ = 0.4924, *P* = 0.0847 > 0.05).

The I/Br ratio (mol/mol) in the groundwater ranged from 0.2 to 3.6 at the site (Fig. S5). The concentration of ^127^I increased with the concentration of boron (Fig. S6a). The concentration of ^129^I in some groundwater samples increased with increasing boron content, while it did not increase in other samples (Fig. S6b). The ^129^I/^127^I ratio showed no correlation with the boron concentration (*R*^2^ = 0.0278, *P* = 0.67 > 0.05) (Fig. S6c).

**Figure 4:**
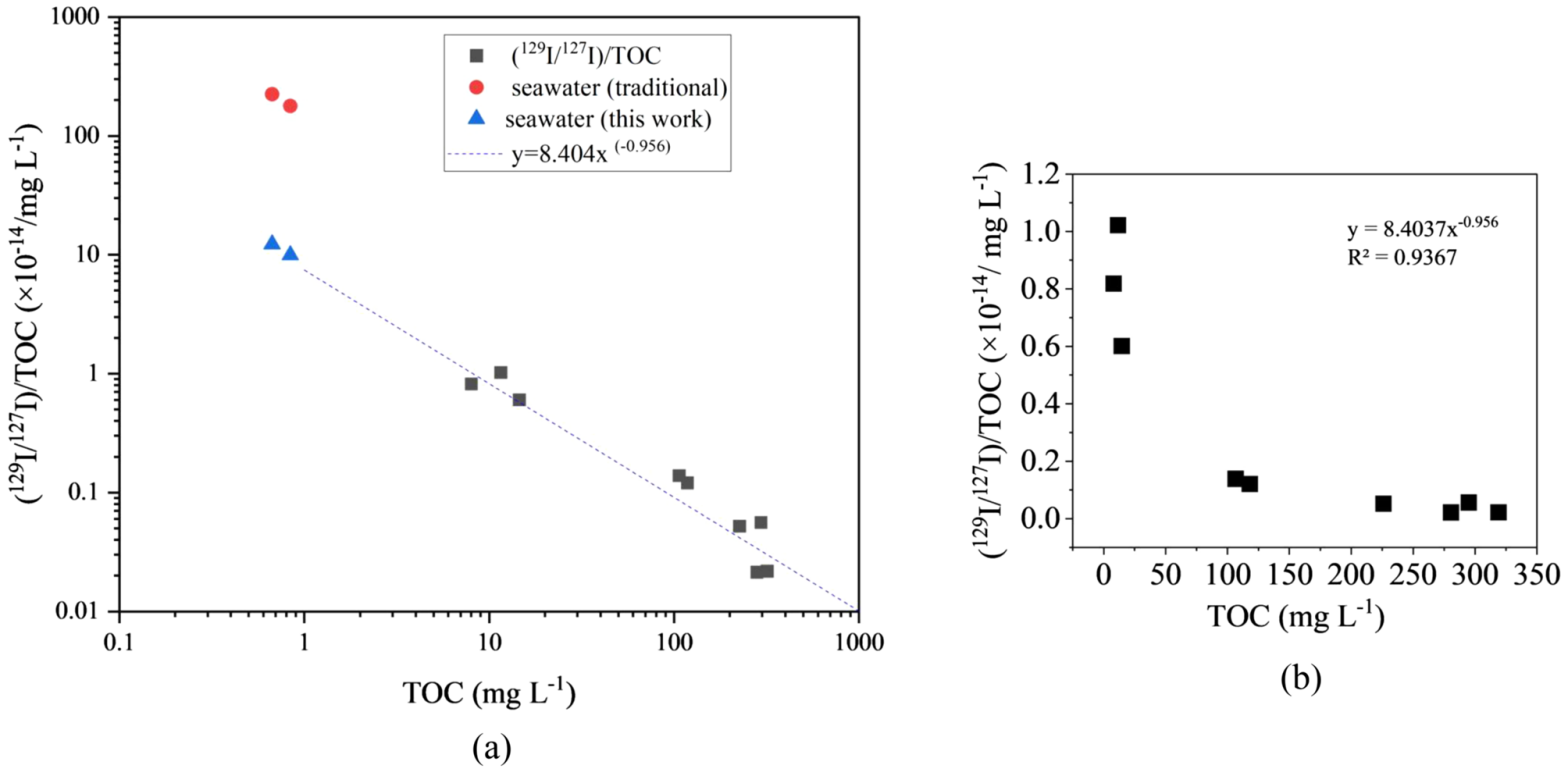
Correlations between the (^129^I/^127^I)/TOC ratio and the concentration of TOC (mg L^-1^) in groundwater. ■: groundwater, ----: regression curve between TOC and (^129^I/^127^I)/TOC in the groundwater, : seawater (Traditional): (^129^I/^127^I)/TOC values of 179 × 10^–14^ and 224 × 10^–14^ calculated from ^129^I/^127^I (150 × 10^–14^, traditional initial ratio estimated by Fehn et al. ^15^) and TOC concentrations in surface seawater of 0.67 and 0.84 mg L^-1^, respectively^38^; : seawater (This study): (^129^I/^127^I)/TOC values of 12.3 × 10^–14^ and 9.93 × 10^–14^ obtained from the regression curve of the groundwater values with TOC concentrations of 0.67 and 0.84 mg L^-1^, respectively.

We normalized ^129^I/^127^I to the concentration of TOC (mg L^-1^) to determine the effect of the addition of old organic matter with low ^129^I/^127^I values. The ratio of (^129^I/^127^I)/TOC decreased with increasing TOC concentration (Fig. 4). The regression curve between ((^129^I/^127^I)/TOC and TOC was ((^129^I/^127^I)/TOC = 8.404 × (TOC) ^-0.956^ (R^2^ = 0.9367), as shown in Fig. 4. We also normalized ^129^I/^127^I to the concentration of boron (mg L^-1^). The ratio of (^129^I/^127^I)/B in the groundwater decreased with the concentration of boron (Fig. S6d). The regression curve between boron and (^129^I/^127^I)/B was (^129^I/^127^I)/B = 10.1 × (B) ^-0.995^, as shown in Fig. S6d (R^2^ = 0.9393).

## Discussion

Assuming that the groundwater residence time estimated from the present ^129^I dating method (50–71 Ma) is correct, the groundwater has risen from a deep underground formation to the ground surface. If the sedimentation process at this site was complete between the Pleistocene and the Pliocene, the estimated groundwater age exhibited large discrepancies between the two prediction methods (groundwater dating by ^81^Kr and ^4^He vs. ^129^I (initial ratio: 1.5 × 10^–12^) groundwater dating) (Table 3). However, the age of groundwater recharge in the Sarabetsu and Yuchi Formations is not older than that in the strata based on our groundwater dating results. This contradiction among the estimated groundwater ages cannot be resolved, even if the traditionally accepted initial ratio of ^129^I/^127^I (1.5 × 10^–12^) is changed to a different value (Table 3). These findings suggest that 1) the groundwater at the site does not well up from deep underground and 2) other factors must be considered to determine the recharge age of groundwater estimated by ^129^I/^127^I.

The iodine dissolved in the groundwater in the Sarabetsu Formation originated from only two sources; the main source was meteoric water that recharged in the last ~ 40 ka, and the other source was dissolution from organic matter in the sedimentary layers that settled offshore between 1 Ma and 0.018 Ma. The iodine in the groundwater in the Yuchi Formation has three origins: (1) seawater and lagoon water from when the coastal sediment was deposited, (2) infiltrated meteoric water from before 0.04 Ma (corresponding to the ^81^Kr age in groundwater at the Upper Yuchi Formation in Table 1), and (3) iodine released from organic matter in the sediment. The concentrations of ^127^I supplied from meteoric water and seawater are only several µg L^-1^ (Yuita et al.^28^) and ~ 58 µg L^-1^ (Elderfield and Truesdale^29^), respectively. Iodine is easily adsorbed on sediments^30,31^ and tends to accumulate in organic matter^32^, cells of marine diatoms^33^ and humic substances^34^. Not all the iodine dissolved into the pore water of the formation; some remained in the sediments as organic iodine during the maturation of organic matter during diagenesis^13^. Considering that the concentration of ^127^I in the groundwater ranged from 10 to 19 mg L^-1^ in the Upper Sarabetsu Formation and from 66 to 84 mg L^-1^ in the Yuchi Formation, the main source of iodine dissolved in the groundwater would be iodine released from organic matter in the sediments.

The organic matter deposited in the sediment has various origins: (1) coeval ocean and (2) old recycled organic matter. We hypothesized that (1) iodine was released into the groundwater through the decomposition of in situ marine organic matter and (2) organic matter with older iodine isotopes contributed to the lower iodine isotopic ratios observed in the groundwater. As the biogenic ratio of I/Br in marine organic materials is greater than 0.04 ± 0.02 (Elderfield and Trudesdale^29^, Pedersen and Price^35^), both the I and Br dissolved in the groundwater could be of biogenic origin. Surface seawater has a boron concentration of 4.5 mg L^-1^; however, boron can be efficiently concentrated into marine products^36,37^ (more than 200 mg L^-1^). The iodine concentration in groundwater increases with increasing boron content, which is released via organic matter decomposition. The results in Figs. S5 and S6 show that the origin of the iodine in the groundwater was organic matter originating from marine organisms (algae, marine plankton) in the sediments. The relationship between the ratio of (^129^I/^127^I)/TOC and TOC shows the effect of the addition of old organic matter with low ^129^I/^127^I values (Fig. 4). This suggests that the groundwater contains recycled organic matter that is older than the sedimentary age of the stratum. Recycled organic matter could be present in any stratum because some recycled organic matter settles on the seafloor due to inflow from river sediments and some is dissolved in seawater.

Here, the primordial ^129^I/^127^I ratio in groundwater is defined as the value when the original seawater is recharged to the surface of the sediment. As the iodine originated from marine products that became concentrated from seawater, the primordial ^129^I/^127^I in the groundwater indicates the value in the sea and lagoon seawater deposited in the Yuchi Formation and Sarabetsu Formation. The concentration of TOC in surface seawater in the open ocean is ~ 58–70 μM (corresponding to a TOC of ~ 0.67–0.84 mg L^-1^) (Figure A2 in Appendix A by Halewood et al.^38^). Here, assuming that the TOC in surface seawater is 0.67–0.84 mg L^-1^, the (^129^I/^127^I)/TOC ratio is (9.9–12.3) × 10^–14^ based on the regression curve in Fig. 4. The ^129^I/^127^I value at TOC concentrations (~ 0.67–0.84 mg L^-1^) corresponding to surface seawater would be 8.3 × 10^–14^ (~ 1 × 10^–13^) based on the regression curve. However, if the TOC and ^129^I/^127^I values in surface seawater are 0.67–0.84 mg L^-1^ and 150 × 10^–14^ (corresponding to the traditional ratios), respectively, the (^129^I/^127^I)/TOC ratio will be (179–224) × 10^–14^ (Fig. 4). This is a significant departure from the extrapolated values from the regression curves between TOC and (^129^I/^127^I)/TOC obtained from the groundwater samples in Fig. 4. Here, when the concentration of boron in surface seawater is 4.5 mg L^-1^, the (^129^I/^127^I)/B ratio estimated from the traditional ^129^I/^127^I model is 33 × 10^–14^, which is plotted well outside the regression curve. However, based on the regression curve in Fig. S6d, when the concentration of boron in the surface seawater is 4.5 mg L^-1^, that of (^129^I/^127^I)/B is 2.26 × 10^–14^. Therefore, the ^129^I/^127^I ratio in the surface seawater when the concentration of boron is 4.5 mg L^-1^ may have been 10.2 × 10^–14^ (~ 1 × 10^–13^). The ^129^I/^127^I value in surface seawater estimated from (^129^I/^127^I)/TOC and TOC is similar to that estimated from (^129^I/^127^I)/B and B. Therefore, the ^129^I/^127^I ratio in seawater when the sediment was deposited between the Yuchi Formation and the Sarabetsu Formation was estimated to be ~ 1 × 10^–13^ based on the regression curve in Fig. 4. Considering that the sedimentation age of the Yuchi Formation at this site corresponds to the late Pliocene (2 Ma), the amount of ^129^I (atoms L^-1^) that decayed out of the total iodine dissolved in the groundwater in the Yuchi Formation to the Sarabetsu Formation is negligible. Therefore, the value of (16.5 ± 0.5) × 10^–14^ (~ 2 × 10^–13^) for the ^129^I/^127^I ratio in the groundwater in the Lower Yuchi Formation includes the primordial ratio.

Assuming that the primordial ratio is ~ 1 × 10^–13^ (8 × 10^–14^ ~ 2 × 10^–13^), the ^129^I/^127^I ratio in seawater is almost the same as the primordial ratio. As the ^129^I/^127^I ratio in the groundwater is almost in the same range of 10^–13^, there is no contradiction with the primordial ratio. Because the average standard deviation (SD) of the ^129^I/^127^I ratio in the groundwater was 14%, the primordial ratio of ^129^I/^127^I agrees with the ratio in groundwater with recharge ages up to 5 Ma within the margin of error. The age of groundwater originating from fossil seawater in the Pleistocene stratum in the Kanto gas field is less than 1 Ma, as estimated by ^36^Cl and ^4^He groundwater dating^2^. The ^129^I/^127^I ratio in groundwater originating from fossil seawater in the Kanto gas field^1^ is ~ 2 × 10^–13^. The ratio for the fossil seawater is similar to our estimated primordial ratio for the seawater.

To obtain accurate groundwater ages in coastal sedimentary areas based on the ^129^I/^127^I ratio in groundwater, the following values need to be clarified: the primordial ratio in the original groundwater recharged in the stratum, the ^129^I/^127^I ratios in the endmembers of the old organic matter and the corresponding abundance ratios. Even the ^129^I/^127^I ratio in recycled organic matter older than the sedimentary age of the stratum will not be constant because old recycled organic matter also contains iodine of various ages. Until multiple endmembers for ^129^I/^127^I in old recycled organic matter are clarified, accurate groundwater dating based on the ^129^I/^127^I index cannot be applied to coastal sedimentary areas. As an alternative to ^129^I/^127^I dating, groundwater dating may be possible with ^129^I-^129^Xe in the future for groundwater with a residence time of tens to 100 Ma.

## Conclusions

The groundwater age estimated from ^129^I groundwater dating using the present accepted initial ^129^I/^127^I ratio of 1.5 × 10^–12^ was more than three orders of magnitude higher than the ages estimated from other (^81^Kr, ^14^C, and ^4^He) dating methods. The ^129^I/^127^I ratio in the groundwater ranged from 6 × 10^–14^ to 2 × 10^–13^, suggesting that iodine in organic matter of various ages was released into the groundwater in the stratum. We propose that the primordial ^129^I/^127^I ratio in fossil seawater is ~ 1 × 10^–13^ (8 × 10^–14^ ~ 2 × 10^–13^). According to the proposed primordial ^129^I/^127^I ratio, all the groundwater ages are consistent (within 5 Ma).

## Methods

### Field site

The boreholes at the investigation site are located in Hamasato on the coast of Hokkaido, Japan, as shown in Fig. 1. The drilling location was 300 m inland from the coastline at an elevation of approximately 5.2 m. Figure 1b shows the geological cross-sectional map of the site. The detailed geological and porewater information for the survey site was described in a previous study^39,40^. In brief, the sediment at the observation point has the following geological structure from a depth of 1200 m to the surface: (1) The Yuchi Formation, which consists of shallow marine sediment, was deposited from a depth of 2 km to 471.5 m during a period from 2.3 Ma or younger in the late Pliocene under an offshore environment. (2) The Sarabestu Formation has overlain the Yuchi Formation since 1.3 Ma in the early Pleistocene, and the site changed from a bay to a lagoon environment. (3) The Sarabestu Formation continued to be deposited in the period from 0.8 to 0.15 Ma (interglacial) in the Middle Pleistocene. The sedimentary environment of the site changed from a lagoon to a river environment. (4) Alluvium was deposited since approximately 10 ka at a thickness of 85 m. That is, at this site, meteoric water started to recharge vertically from the surface alluvium between 0.8 Ma and 0.15 Ma when the lagoon changed to a freshwater body. The pore water at this site mainly changed from fossil seawater to freshwater in the present age at depths from the surface to the Sarabetsu Formation, suggesting that meteoric water displaced the fossil seawater that had existed from the surface to the Sarabetsu Formation and penetrated since 0.8 Ma from the alluvium to the Sarabetsu Formation (Fig. S1). Although fossil seawater remains in the Yuchi Formation, the pore water in the upper part of the Yuchi Formation is a mixture of meteoric water and fossil seawater. The groundwater in the present alluvium and the Sarabetsu Formation consists of fresh water, while the groundwater in the upper part of the Yuchi Formation is a mixture of meteoric and fossil seawater, and the Cl concentration in the Yuchi Formation (− 1200 m) is between 10,000 mg L^-1^ and 17,000 mg L^-1^.

### Sampling of deep groundwater at the site

Groundwater samples were collected from three packed-off borehole sections by pumping after drilling to depths of 1200 m (DD-1 borehole), 100 m (DD-2 borehole) and 360 m (DD-4) (Fig. S3). The screen depths were 214–215 m, 306–307 m, 476–477 m, 613–614 m, 715–716 m, 943–944 m, and 1143–1144 m for the DD-1 borehole; 90.7–99.7 m for the DD-2 borehole; and 337–348 m for the DD-4 borehole, as shown in Fig. 1c. The groundwater samples were collected within the perforation interval using an upper packer and lower packer from boreholes drilled to depths ranging from 90.7 to 99.7 m and 214 to 215 m for the Upper Sarabetsu Formation; from 306 to 307 m and 337 to 348 m for the Lower Sarabetsu Formation; from 476 to 477 m, 613 to 614 m, and 715 m to 716 m for the Upper Yuchi Formation; and from 943 to 944 m and 1143 to 1144 m for the Lower Yuchi Formation. Groundwater for radio-Kr, ^14^C, radioiodine, and other chemical analyses was pumped and sampled above ground (Fig. S3a). After sampling, the noble gases present in the groundwater were collected into copper tubes in situ (Fig. S3b,c). The in situ sampler mainly consisted of a piston sampler, copper tubes (three vertically connected) and a check valve. The piston sampler was pressurized with ethanol and lowered to the target depth of groundwater. At the target depth, the pressure of the piston sampler was reduced, and the groundwater was passed through the copper tube. After sampling was complete, the piston sampler was pressurized to close the check valve and raised to the ground. The field campaign was held in 2017–2018.

### ^129^I

Groundwater samples were collected in plastic bottles. The method for the extraction and purification of iodine was as follows. The dissolved iodine was oxidized to I_2_ and was then separated from the sample water into 10 ml of organic solvent (CCl_4_ or hexane). The solvent was separated from the sample water, and then 10 ml of 0.1 M Na_2_SO_3_ or NaHSO_3_ was added to the solvent to extract I^−^ into the Na_2_SO_3_ or NaHSO_3_ solution. The Na_2_SO_3_ or NaHSO_3_ solution was separated from the solvent, and 0.1 ml of concentrated HNO_3_ was added to the solution. A 0.1 ml portion of 1 M AgNO_3_ was then added to the solution, and the solution was agitated. Precipitation was allowed to occur for 30 min before the mixture was centrifuged for 5 min at 3000 rpm. The AgI precipitate was separated from the solution. The AgI precipitate was rinsed with 5 ml of ultrapure water to yield a pure AgI sample, which was then dried in an electric oven at 80 °C for 12 h. The ^129^I/^127^I isotopic ratio in the AgI sample was measured with an accelerator mass spectrometer (Ottawa University and The University of Tokyo). When the amount of AgI was estimated to be less than 1 mg, an isotope dilution method was applied. The ^129^I/^127^I isotopic ratio of the carrier for isotope dilution was 3 ± 1 × 10^–14^. The ^127^I concentration in the groundwater was measured by ICP‒MS.

### ^81^Kr

Dissolved gas samples were separated by a hollow fiber method with water replacement in the field^41,42^ to collect the necessary samples for ^81^Kr dating^9^. Depending on the methane content of the dissolved gas, the extracted Kr values ranged from 0.2 to 10 µL STP. The relative isotopic abundances of both ^81^Kr and ^85^Kr were measured by atom trap trace analysis (ATTA)^9^. Since the half-life of ^85^Kr is only 10.7 a, any presence of ^85^Kr is an indication of modern air contamination, which could be introduced in wellheads, pumping systems or sample treatment processes. Assuming that the contamination is from local air during sampling, the ^85^Kr activity can be used to make contamination corrections to the measured ^81^Kr abundance (R_81_meas_). The ^81^Kr abundance (R_81_corr_) after correction can be expressed as follows:

1 $${\text{R}}_{{81\_{\text{corr}}}} = \, \left( {{\text{R}}_{{81\_{\text{meas}}}} - 100 \times \alpha } \right)/\left( {1 \, - \, \alpha } \right)$$

where α is the fraction of Kr from modern contaminants. The contamination fraction α can be determined using the measured ^85^Kr activity (R_85_meas_) and the ^85^Kr activity of the local air at the sampling site:

2 $$\alpha = {\text{R}}_{{85\_{\text{meas}}}} /\left( {{\text{R}}_{{85\_{\text{atm}}}} } \right)$$

By combining Eqs. (1) and (2), the corrected ^81^Kr abundance (R_81_corr_) can be calculated. The value of R_85_atm_ is assumed to be 75 dmp/cc_Kr_^43–45^.

### Analytical methods for the chemical characterization of groundwater

We measured stable hydrogen and oxygen isotope ratios (δD and δ^18^O), as well as the concentrations of eight major dissolved ions (Na^+^, K^+^, Ca^2+^, Mg^2+^, Cl^−^, SO_4_^2−^, NO_3_^−^) and dissolved trace elements (total amount of Br, I, and B) in the samples. The major ion and trace element concentrations were measured by ion chromatography (IC881, Metrohm Co., Tokyo, Japan) and ICP-MS (inductively coupled plasma‒mass spectrometry, 7500 CE, Agilent Technologies Inc., CA, USA), respectively. δD and δ^18^O values were measured by the cavity ring-down method (IWA-35EP, isotope ratio mass spectrometry, Los Gatos Research, San Jose, CA, USA) and Iso-Prime (GV Instruments, Manchester, UK), respectively. The groundwater was distilled for ^3^H analysis and then subjected to electrolytic enrichment of tritium. The enriched tritium samples were mixed with a scintillation cocktail. The radioactivity of the tritium in the samples was measured by a liquid scintillation counter (LSC-LB5, Aloka, Co. Ltd.). Other groundwater dating data collected at the borehole site were estimated from noble gases (He, Ne, Ar, Kr, and Xe) and ^14^C^24^. The ^14^CO_2_ in the groundwater was separated by gas stripping^46^ and then further purified to obtain ^14^C. The ^14^C/C ratio was measured by accelerator mass spectrometry (AMS) (Beta Analytical Co. Ltd., USA). TOC was measured with a nondispersive infrared gas analyzer. δ^13^C was measured by a stable isotope mass spectrometer (Beta Analytical Co. Ltd., USA).

### Supplementary Information

Supplementary Information.

## Data Availability

All data generated or analyzed during this study are included in this published article (and its Supplementary Information files).
