# Author Correction: Feasibility of ^129^I groundwater dating calibrated by both ^81^Kr and ^4^He for the assessment of deep geological repositories in Japan

**DOI:** 10.1038/s41598-024-70487-3

**Published:** 2024-08-27

**Authors:** Tomoko Ohta, Takuma Hasegawa, Wei Jiang, Guo Min Yang, Zheng‑Tian Lu, Yasunori Mahara

**Affiliations:** 1 Civil Engineering Research Laboratory (Sustainable System Research Laboratory), Central Research Institute of Electric Power Industry, Abiko, Chiba 270-1176 Japan; 2 Department of Nuclear Technology, Nagaoka University of Technology, Kamitomioka, Nagaoka, Niigata 940-2188 Japan; 3 CAS Center for Excellence in Quantum Information and Quantum Physics, School of Physical Sciences, University of Science and Technology of China, Hefei, 230026 China; 4 Hefei National Laboratory, University of Science and Technology of China, Hefei, 230088 China; 5 Kyoto University, Sakyo-Ku, Kyoto, 606-8501 Japan

Correction to: *Scientific Reports* 10.1038/s41598-024-66250-3, published online 08 July 2024

The Acknowledgements section in the original version of this Article was incomplete.

“This study was conducted under a contract with the Ministry of Economy, Trade and Industry (METI) as part of the R&D support program titled “Development of enhancing the disposal system in the coastal region (2019Fy)” (Grant Number JPJ007597). The original idea and basic research for determining the primordial ratio of ^129^I/^127^I in groundwater were established in the period from 2008–2010 (supported by a Grant-in Aid for Scientific Research A, KAKENHI number 20241006), and basic research was partly supported by KAKENHI Grant Numbers 24686098 and 20H02674. We thank the National Institute of Advanced Industrial Science and Technology (Drs. Ono, Machida, Ikawa, Marui, and Matsumoto) for their cooperation and CRIEPI members (Dr. Tomioka and Mr. Okamoto) for groundwater sampling.”

now reads:

“This study was conducted under a contract with the Ministry of Economy, Trade and Industry (METI) as part of the R&D support program titled “Development of enhancing the disposal system in the coastal region (2019Fy)” (Grant Number JPJ007597). The original idea and basic research for determining the primordial ratio of ^129^I/^127^I in groundwater were established in the period from 2008–2010 (supported by a Grant-in Aid for Scientific Research A, KAKENHI number 20241006), and basic research was partly supported by KAKENHI Grant Numbers 24686098 and 20H02674. 

This work has been supported by the Innovation Program for Quantum Science and Technology (2021ZD0303101), the National Natural Science Foundation of China (41727901). We thank the National Institute of Advanced Industrial Science and Technology (Drs. Ono, Machida, Ikawa, Marui, and Matsumoto) for their cooperation and CRIEPI members (Dr. Tomioka and Mr. Okamoto) for groundwater sampling.”

The original Article has been corrected.

