# Supplementary material for: Feasibility of 129I groundwater dating calibrated by both 81Kr and 4He for the assessment of deep geological repositories in Japan
Source: Sci Rep. 2024 Jul 8;14:15688. doi: 10.1038/s41598-024-66250-3 (PMC11231164; doi:10.1038/s41598-024-66250-3)
Supplement: Supplementary file 1 — Supplementary Information. [file 41598_2024_66250_MOESM1_ESM.pdf]

Supplementary Information

Feasibility of ^{129}I groundwater dating calibrated by both ^{81}Kr and ^{4}He for the assessment of deep geological repositories in Japan

Tomoko Ohta[1,2,*], Takuma Hasegawa[1], Wei Jiang[3,4], Guo Min Yang[3,4], Zheng-Tian Lu[3,4], Yasunori Mahara[5]

1. Civil Engineering Research Laboratory (Sustainable System Research Laboratory), Central Research Institute of Electric Power Industry, Abiko, Chiba, 270-1176, Japan

2. Department of Nuclear Technology, Nagaoka University of Technology, Kamitomioka, Nagaoka, Niigata, 940-2188, Japan

3. CAS Center for Excellence in Quantum Information and Quantum Physics, School of Physical Sciences, University of Science and Technology of China, Hefei, 230026, China

4. Hefei National Laboratory, University of Science and Technology of China, Hefei, 230088, China

5. Kyoto University, Sakyo-ku, Kyoto city, Kyoto 606-8501, Japan

*Corresponding author: tomoohta@vos.nagaokaut.ac.jp

Figure captions

Table S1 Concentrations of tritium, ^{14}C, TOC, δD, $δ^{18}O$, and trace elements

Figure S1 Sedimentary age and geohistorical map of the sampling site.
***reorganization quoted from Ikawa et al.** [39]

Figure S2 Concentrations of major ions in the groundwater
(a) Hexa-diagram, (b) Piper diagram

Figure S3 Schematic drawings of groundwater sampling at the sampling site
(a) Ground sampling, (b) overview of in situ sampling, (c) detailed in situ sampling

Figure S4 Correlation between iodine and TOC in the groundwater.
■: ^{127}I, ●: ^{129}I

Figure S5 Correlation between I/Br and 1/Cl.
■: **groundwater,** ●: **seawater, pink area: biogenic ratio of I/Br (≥0.04 ± 0.02)**

Figure S6 Correlation between iodine and boron.
(a) concentration of ^{127}I and B, (b) concentration of ^{129}I and B , T: only two points have low ^{129}I concentrations, A: regression curve excluding two points of T, (c) $^{129}I/^{127}I$ ratio and B, (d) $^{129}I/^{127}I/B$ ratio and B, ■: groundwater, ●: surface seawater estimated from $^{129}I/^{127}I$ value (150×10^{-14})[15] and concentration of B in surface seawater (4.5 mg L^{-1})

Table S1*

Geology	Depth	^{3}H*,**	TOC	TC	TIC	δ^{13}C	^{14}C*	δD*	δ^{18}O*	Br*	I/Br	Cl*	1/Cl	B
	m	TU	mg L^{-1}	mg L^{-1}	mg L^{-1}		pMC	‰	‰	mg L^{-1}	mol mol^{-1}	mg L^{-1}	mol^{-1}	mg L^{-1}
Upper Sarabetsu F.	90.7 - 99.7	DL	12	100	90		14 ± 0.1	-81	-11	1.7	3.6	150	233	0.5
	90.7 - 99.7	DL		120	100		11 ± 0.1	-82	-12	1.8		160	219	
	90.7 - 99.7	DL		100	91	-1.6	9 ± 0.1	-80	-12	1.7		160	219	
	214 - 215	DL	15	110	92	-0.5	2 ± 0.0	-80	-12	5.8	2.0	570	61	0.9
Lower Sarabetsu F.	306 - 307	DL	10.2	61	47		22 ± 0.1	-69	-11	1.3		130	269	
	306 - 307	DL	8.0	46	36	-13.8	15 ± 0.1	-68	-10	2.9	0.28	280	125	0.5
	337 - 348	DL	107	42	34	-8.9	15 ± 0.1	-69	-11	1.6	0.34	150	233	0.3
Upper Yuchi F.	476 - 477	DL	118	170	130	+0.8	12 ± 0.1	-70	-10	18	1.7	1600	22	3.8
	613 - 614	DL	319	480	420		3 ± 0.1	-46	-6	98	0.42	8500	4	3.8
	613 - 614	DL		540	420	+2.7	0.4	-45	-6	99		9300	4	
	715 - 716	DL	281	1100	730	+1.1	1 ± 0.0	-13	1	180	0.29	17000	2	11
Lower Yuchi F.	943 - 944	DL	226	900	710		6 ± 0.0	-11	1	190	0.22	17000	2	11.8
	943 - 944	DL		900	620	-0.2	1 ± 0.1	-11	1	190		17000	2	
	943 - 944	DL		870	640		1 ± 0.0	-10	2	190		17000	2	
	943 - 944	DL		790	530	-0.3	0.4	-10	2	180		17000	2	
	1143 - 1144	DL	295	980	710		3 ± 0.1	-14	2	170	0.31	16000	2	13
Sea water***			0.67-0.84****							67	0.00055	19350	1.8	4.5

*Hasegawa et al.[24], **DL: 0.1 TU, ***Br, Cl in seawater: Mahara et al.[1], I: Elderfield and Truesdale[29], **** Halewood et al.[38]

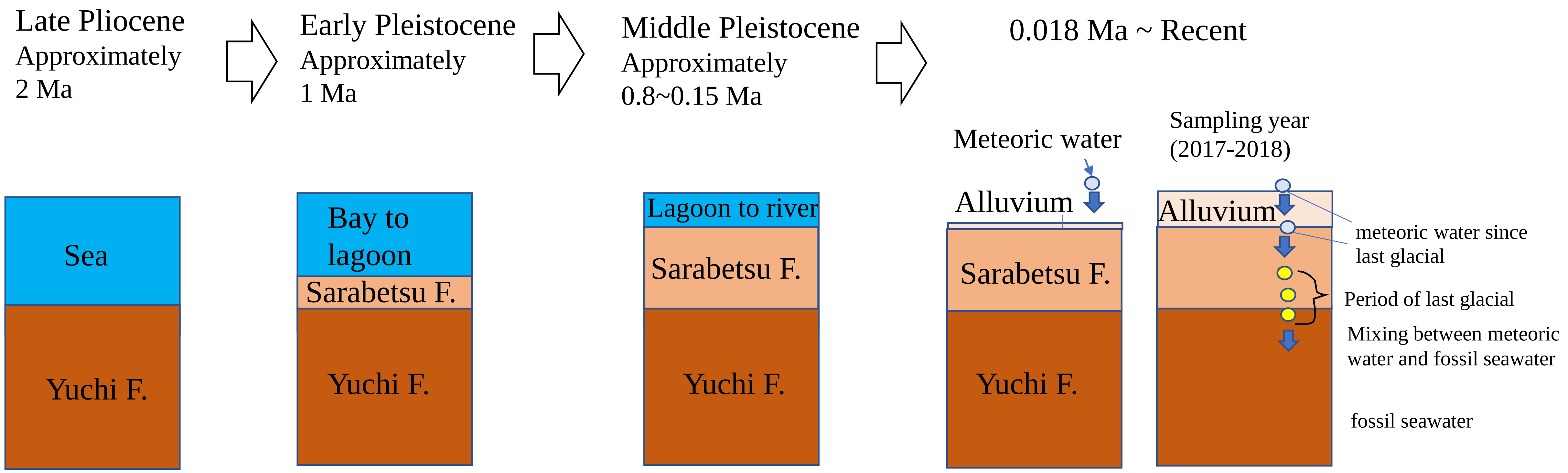

***reorganization quoted from Ikawa et al.[39]**

Fig. S1

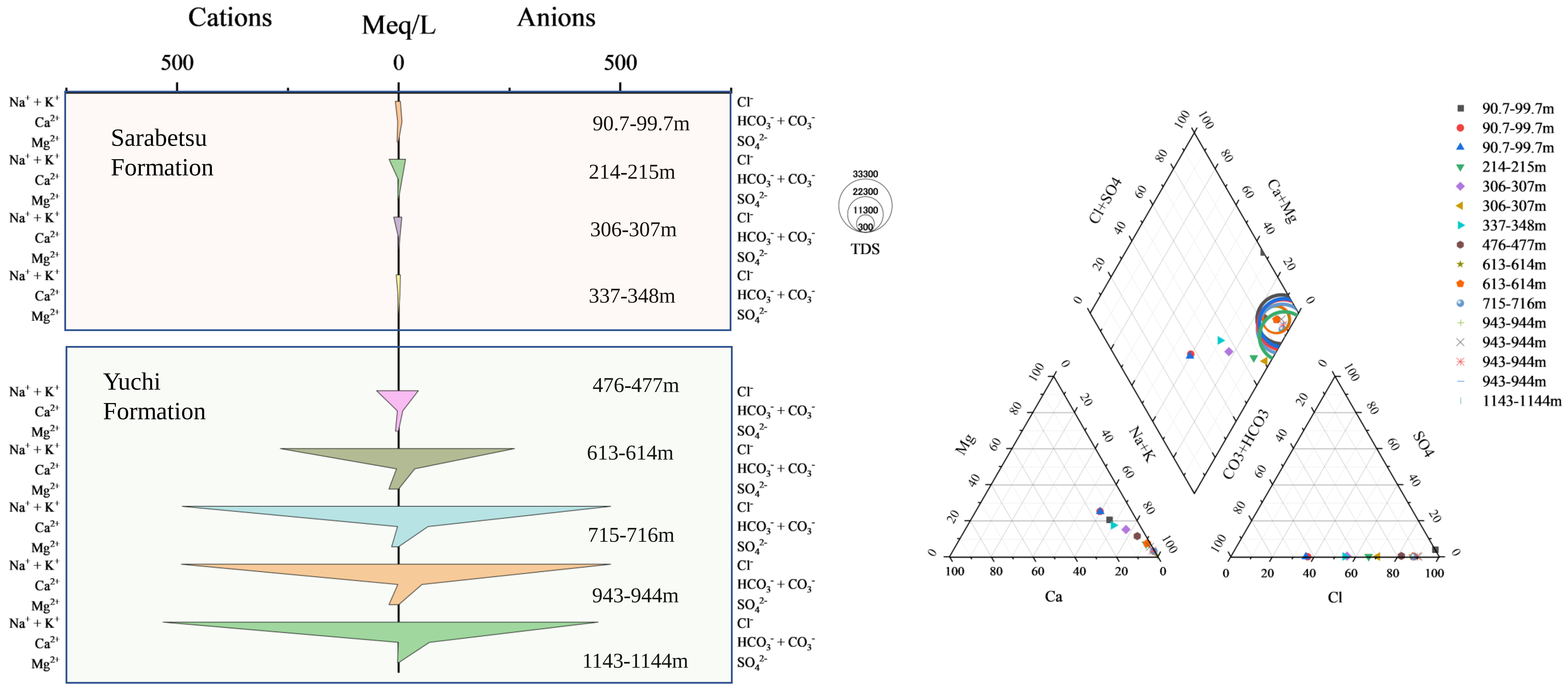

Cations
Meq/L
Anions
500
0
500
Sarabetsu Formation
Yuchi Formation
90.7-99.7m
214-215m
306-307m
337-348m
476-477m
613-614m
715-716m
943-944m
1143-1144m
$Na^+ + K^+$
Ca^{2+}
Mg^{2+}
Cl^-
$HCO_3^- + CO_3^-$
SO_4^{2-}
33300
22300
11300
300
TDS
Cl+SO4
Ca+Mg
Mg
Na+K
Ca
CO3+HCO3
SO4
Cl
90.7-99.7m
90.7-99.7m
90.7-99.7m
214-215m
306-307m
306-307m
337-348m
476-477m
613-614m
613-614m
715-716m
943-944m
943-944m
943-944m
943-944m
1143-1144m

(a)

(b)

Fig. S2

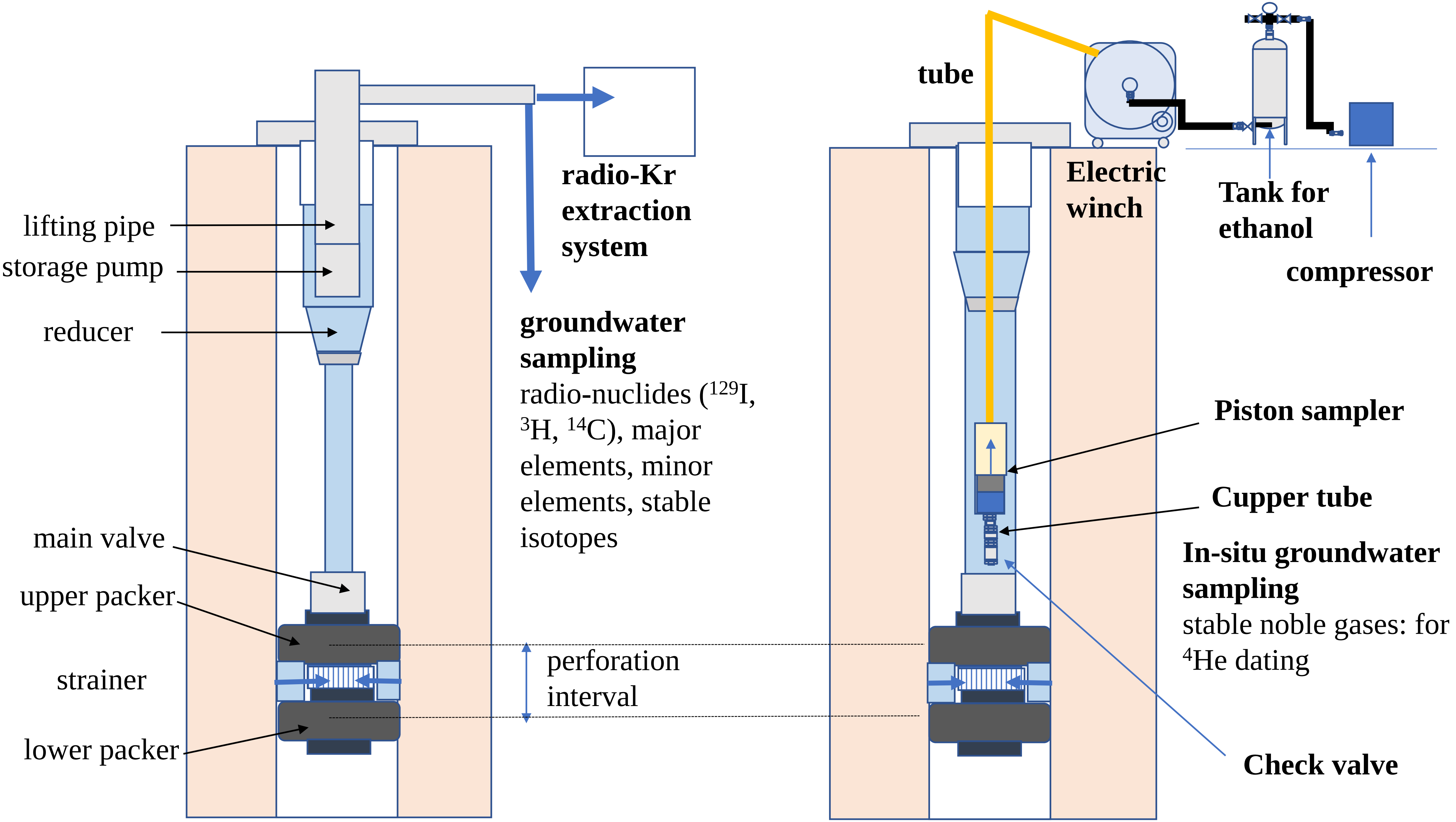
lifting pipe
storage pump
reducer
main valve
upper packer
strainer
lower packer
radio-Kr extraction system
groundwater sampling
radio-nuclides (129I, 3H, 14C), major elements, minor elements, stable isotopes
perforation interval
tube
Electric winch
Tank for ethanol
compressor
Piston sampler
Cupper tube
In-situ groundwater sampling
stable noble gases: for 4He dating
Check valve

(a) (b)

Fig. S3

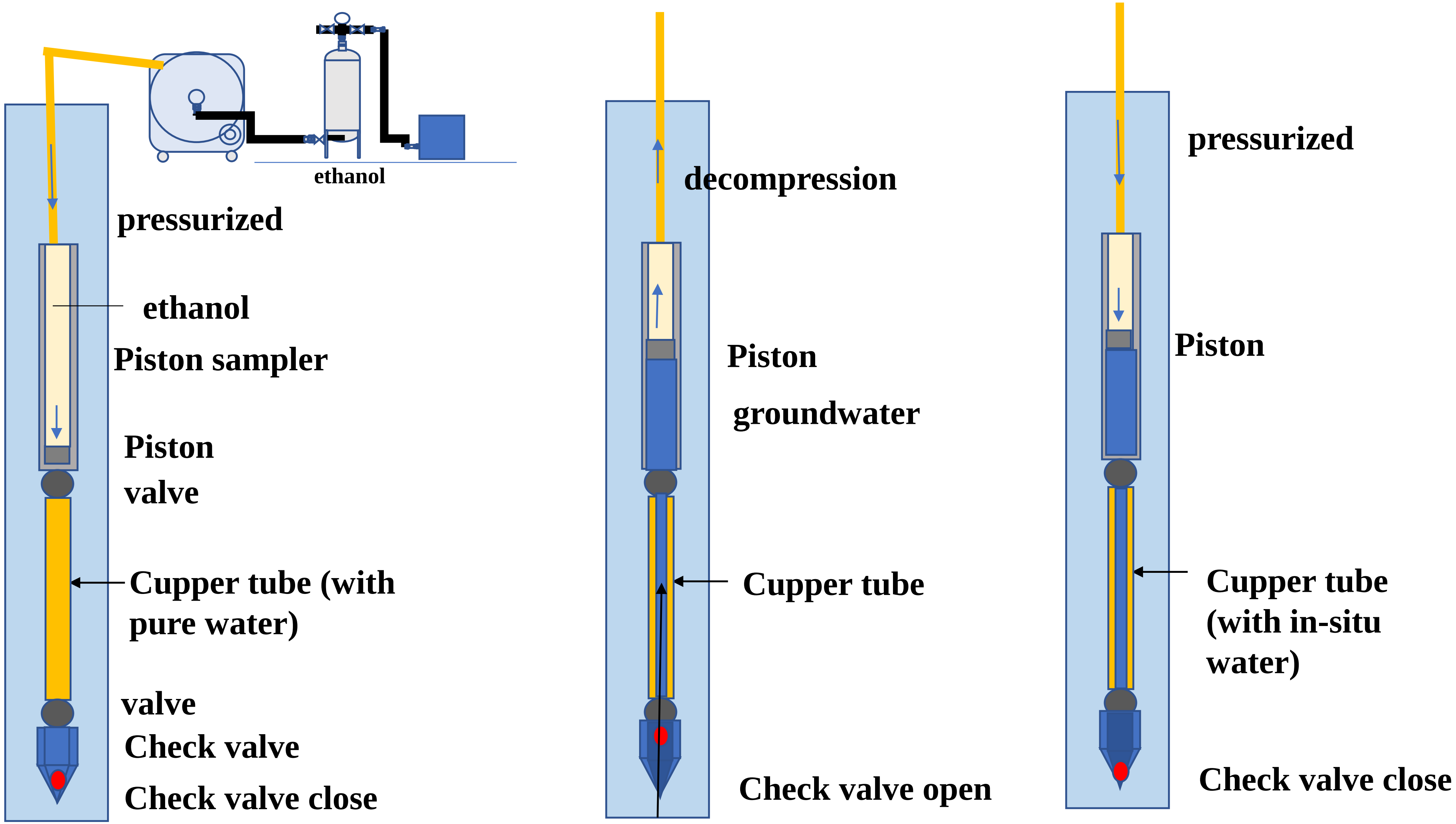

①The process of lowering sampler with a winch ②In-situ sampling ③ after in-situ sampling

(c)

Fig. S3

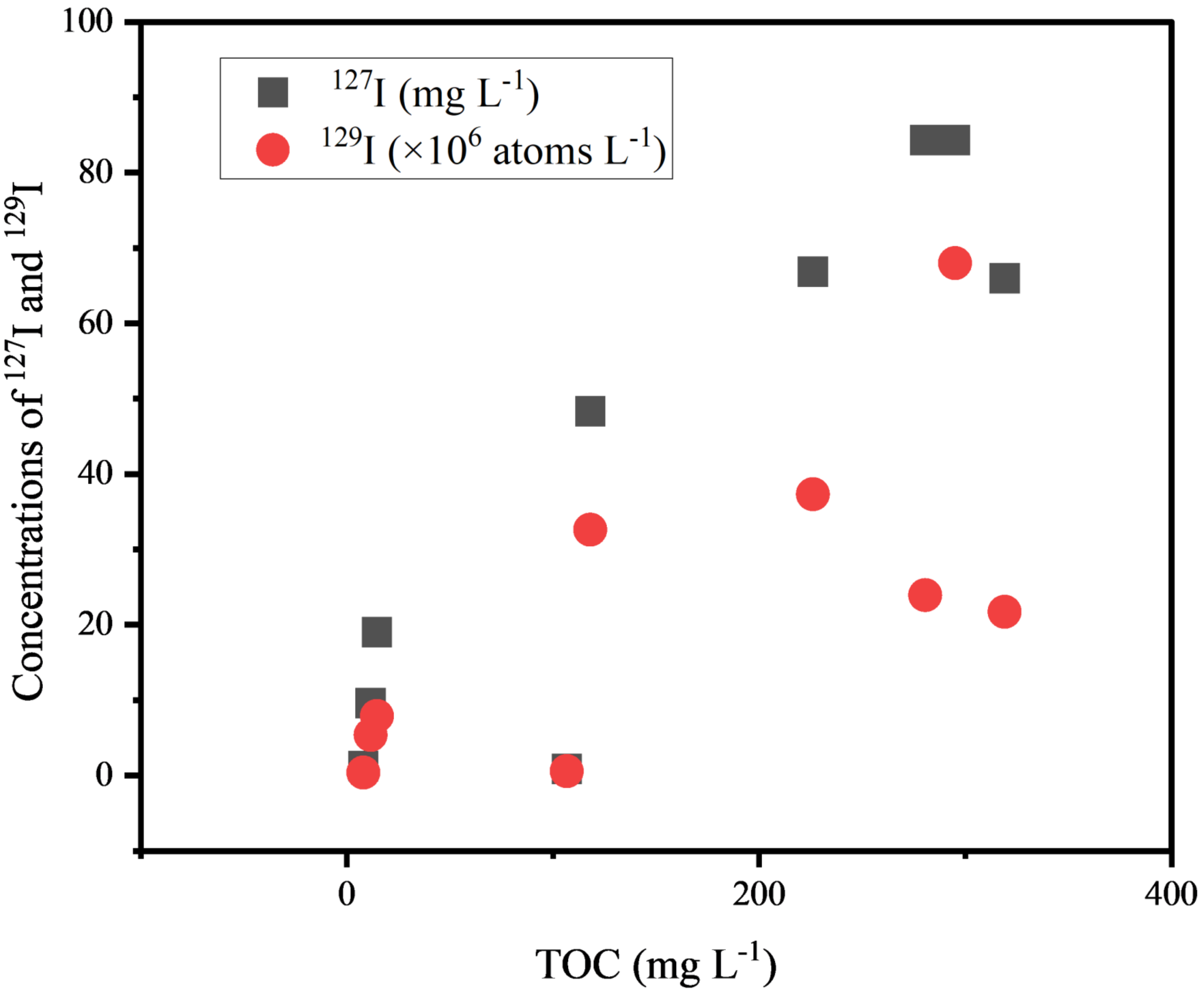

100
80
60
40
20
0
Concentrations of ^{127}I and ^{129}I
^{127}I (mg L^{-1})
^{129}I (×10^6 atoms L^{-1})
0
200
400
TOC (mg L^{-1})

Fig. S4

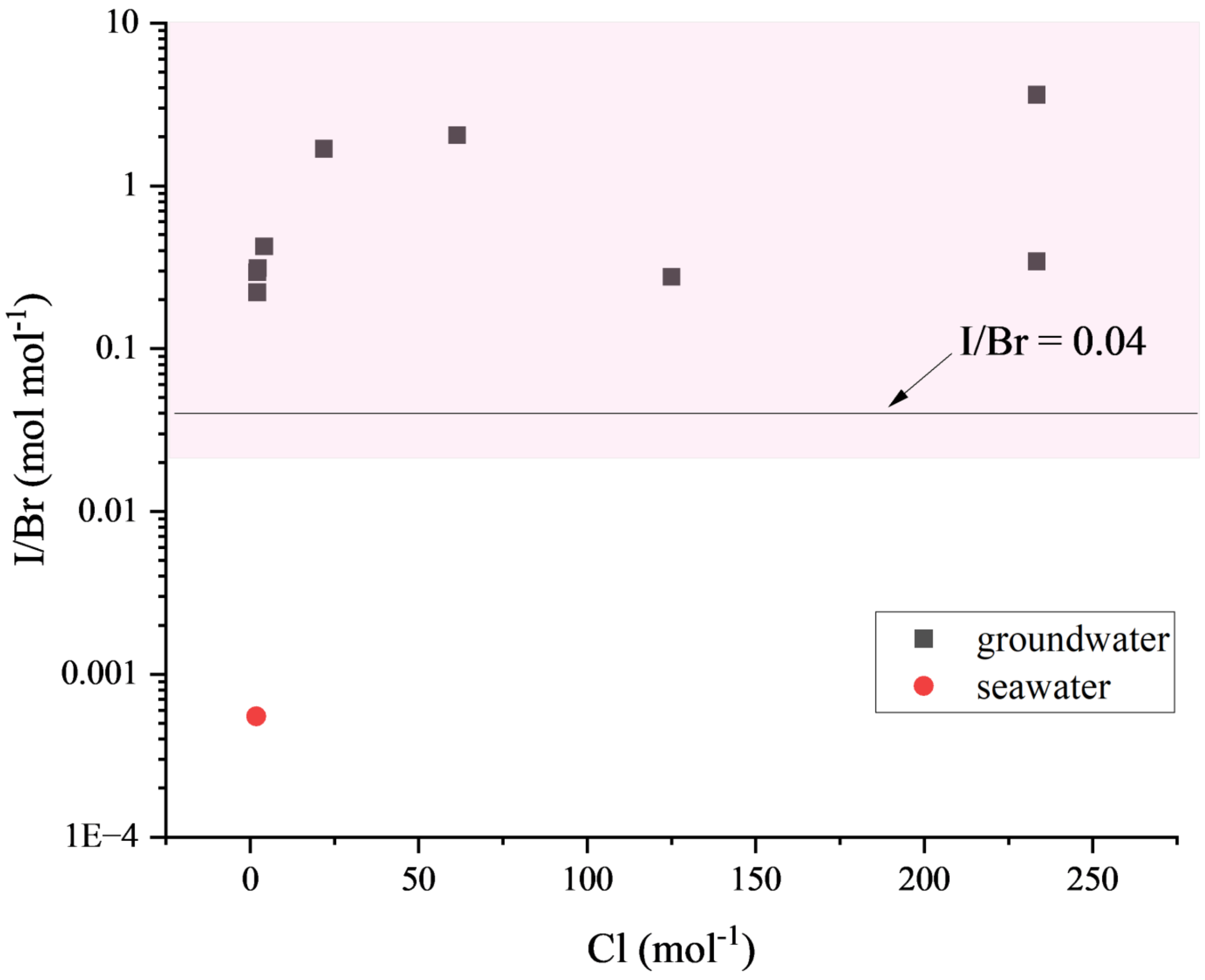
10
1
0.1
0.01
0.001
1E−4
I/Br (mol mol-1)
0
50
100
150
200
250
Cl (mol-1)
I/Br = 0.04
groundwater
seawater

Fig. S5

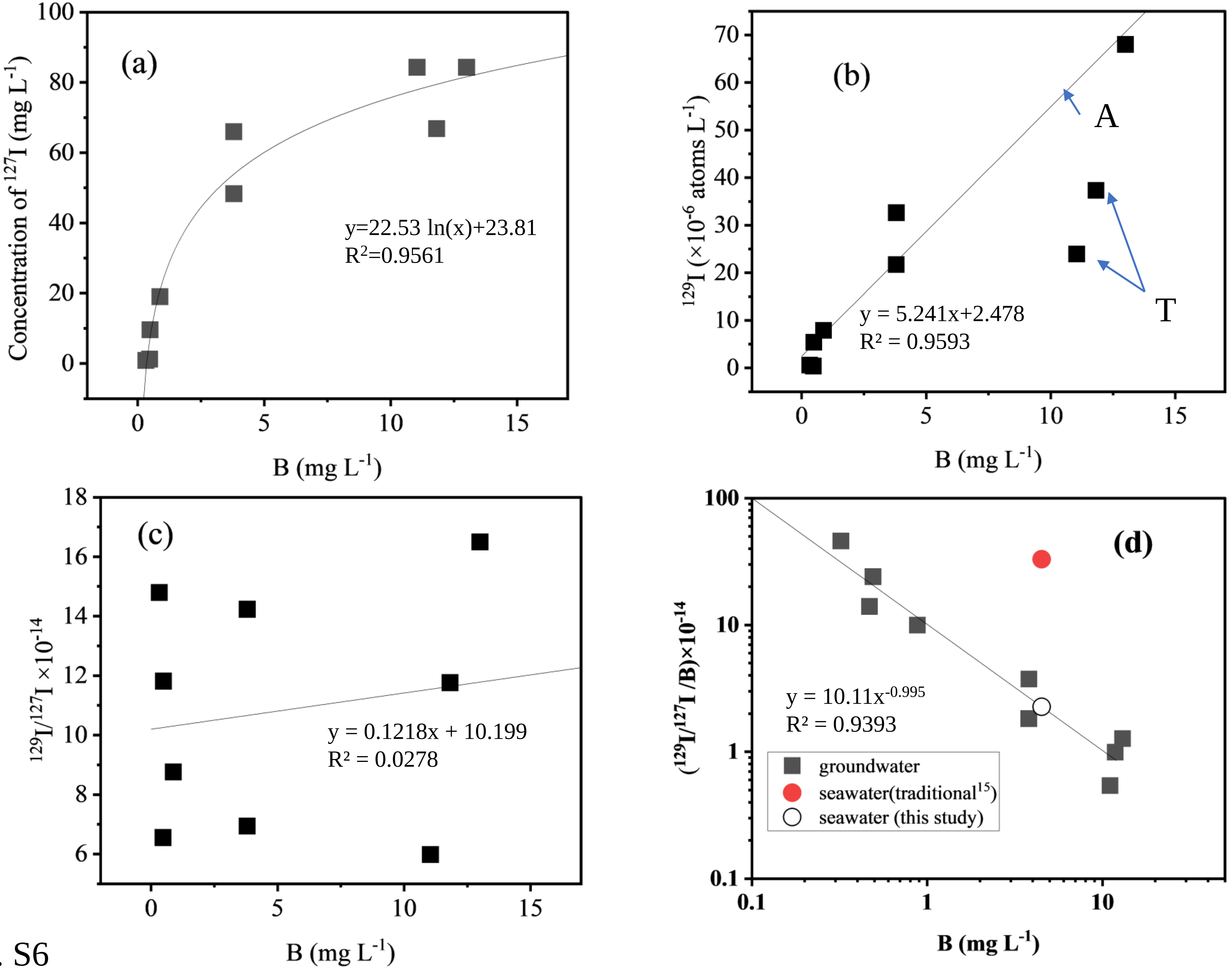
(a)
Concentration of 127I (mg L-1)
y=22.53 ln(x)+23.81
R2=0.9561
B (mg L-1)
(b)
129I (×10-6 atoms L-1)
A
T
y = 5.241x+2.478
R² = 0.9593
B (mg L-1)
(c)
129I/127I ×10-14
y = 0.1218x + 10.199
R² = 0.0278
B (mg L-1)
(d)
(129I/127I /B)×10-14
y = 10.11x-0.995
R² = 0.9393
groundwater
seawater(traditional15)
seawater (this study)
B (mg L-1)

Fig. S6
